# Commentary: A robust data-driven approach identifies four personality types across four large data sets

**DOI:** 10.3389/fdata.2020.00008

**Published:** 2020-02-25

**Authors:** Kentaro Katahira, Yoshihiko Kunisato, Yuichi Yamashita, Shinsuke Suzuki

**Affiliations:** ^1^Department of Psychological and Cognitive Sciences, Graduate School of Informatics, Nagoya University, Nagoya, Japan; ^2^Department of Psychology, Senshu University, Kawasaki, Japan; ^3^Department of Information Medicine, National Institute of Neuroscience, National Center of Neurology and Psychiatry, Tokyo, Japan; ^4^Brain, Mind and Markets Laboratory, Department of Finance, Faculty of Business and Economics, The University of Melbourne, Parkville, VIC, Australia

**Keywords:** personality types, cluster, Gaussian mixture models, skewness, statistical modeling

What kinds of personalities do humans have? Can these personalities be classified into several discrete types? These issues have been of considerable concern as they could potentially provide deeper understanding of the nature of human individuality and mental disorders. Recently, Gerlach et al. (2018) addressed these issues by applying established machine-learning techniques to big data (more than 1.5 million respondents in total). They found four “meaningful clusters” in personality dimensions, suggesting the existence of at least four personality types. Here, we propose an alternative interpretation of their result: a skewed distribution with no cluster structures in personality space can erroneously lead to the seemingly meaningful clusters.

## Distribution of Personality

It is now widely accepted that human personality is characterized by five dimensions (traits or factors), which consist of neuroticism, extraversion, openness, agreeableness, and conscientiousness (Goldberg, 1990). Yet, understanding of how human personalities are distributed in this five-dimensional (5D) space remains elusive. There exist at least two major views: the *dimensional* view and *categorical* view. The dimensional view supposes that the distribution is unimodal and individuals' personalities are continuously distributed in the 5D space. The categorical view posits that there are multiple clusters (dense regions) in personality space (i.e., the distribution is multimodal) and each individual can be classified into one of these clusters. In personality theory, such clusters are referred to as personality “types.” While common analytical tools of personality (e.g., factor analysis) are constructed based on the dimensional view, some researchers have considered the categorical view and claimed the existence of personality types (e.g., Robins et al., 1996).

A recent study by Gerlach et al. (2018) aimed to identify personality types in a highly robust manner based on four large data sets. Their analyses identified four meaningful clusters deemed as personality types. However, in the present study, we suggest that Gerlach et al.'s analysis cannot necessarily exclude the dimensional view. In particular, we demonstrate that a skewed distribution without a cluster structure can lead to spurious clusters that are deemed “meaningful clusters” or “types” by Gerlach et al.'s analysis.

## Procedure of Analysis and Its Pitfall

**Figure 1:**
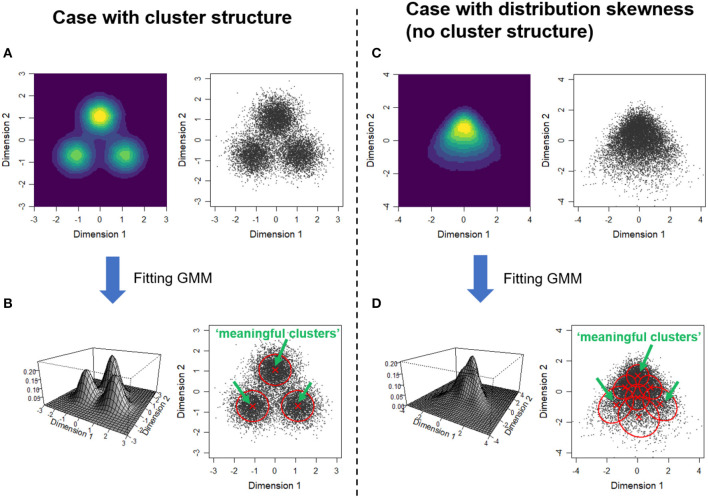
How clustering based on a Gaussian mixture model works for two types of distributions. Comparison of a case with a clear cluster structure **(A,B)** and a case with a skewed distribution **(C,D)**. **(A,C)** The right panel for each shows the scatter plot of synthesized data and the left shows the estimated probability density from the samples. **(B,D)** Result of fitting Gaussian mixture models. The left panel shows the probability density functions of the fitted GMM. The right panel indicates the one standard deviation contours and the centers of the estimated Gaussian components (the scatter plots that are identical with the above panels are shown). The Gaussian components deemed “meaningful clusters” by Gerlach et al.'s procedure are indicated by green arrows (see Supplementary Text 2 and Supplementary Figure 1 for details).

The core part of Gerlach et al.'s analysis is fitting Gaussian mixture models (GMM) to the five factor scores that provide the positions of individuals in the 5D space (the procedure adopted in Gerlach et al. is briefly described in Supplementary Text 1). GMM represents a given distribution by weighted sum of a finite number of Gaussian (normal) distributions. If there are indeed cluster structures and each cluster can be represented by single Gaussian distribution, each Gaussian component may correspond to a single cluster (Figures 1A,B). To examine whether each Gaussian component is a truly meaningful cluster, they performed a statistical test based on the null model that assumes the five factors are distributed independently of each other. As a result, they identified four Gaussian components as meaningful clusters.

However, even if the target distribution is unimodal and there is no cluster structure, similar results (i.e., emergence of meaningful clusters) can be obtained when the distribution has skewness (Figures 1C,D). In the simulation, we applied the procedure to 2D data artificially generated from a unimodal, skewed distribution (see Supplementary Text 2). The GMM has a property that can fit a non-Gaussian distribution by combining multiple Gaussian components (Roeder and Wasserman, 1997; Bauer and Curran, 2004). In this case, the best fitted GMM had seven components to represent the skewed distribution (Figure 1D). Among these components, three were deemed “meaningful clusters” given that the density of each component center was significantly higher than the null model (Supplementary Figure 1). It should be noted that, in addition to the skewness of marginal distribution (distribution of each variable where the other variable is marginalized out), the dependence among factors is necessary for the emergence of spurious meaningful clusters. This is because the null model has the same marginals as the original distribution (as indicated by the comparison of Supplementary Figures 1B,C).

This mechanism could have influenced Gerlach et al.'s results. The distributions of their factor scores were found to be skewed (Supplementary Text 3), and to some extent there appears to be a statistical dependence between different factors, i.e., the shapes of 2D joint distributions of two factors differ from the product of the marginals (Supplementary Figure 2 of Gerlach et al.). Based on these considerations, we suggest that the results of Gerlach et al. do not necessarily reflect the cluster structures and instead could reflect skewness of the distribution. The distribution of factor scores can be skewed, for example, by range restriction (discretization) of responses to questionnaire-items (see Rice and Richardson, 2014). The dependence among factor scores can arise due to a rotation procedure in the factor analysis; non-linear dependence between dimensions arises by rotating non-Gaussian variables (Hyvärinen and Oja, 2000).

Our discussion is closely related to another commentary on Gerlach et al. (2018) by Freudenstein et al. (2019). By reanalyzing the Johnson-300 data set (Johnson, 2014), Freudenstein et al. (2019) pointed out that only less than half (42%) of the respondents was classified into four meaningful clusters. The mechanism that we suggested provides a natural explanation to this result. That is, if meaningful clusters just represent the edge of the skewed distribution rather than a higher density region in the fitted model (as in Figure 1D), the majority of the samples are not necessarily classified into such clusters. Indeed, only 45.5% of the samples in Figure 1D are classified into one of the three “meaningful clusters” (Supplementary Figure 1G).

In conclusion, we have demonstrated the possibility that the skewness of the distribution can influence the personality types reported by Gerlach et al. (2018), although we did not formally evaluate how much their results indeed suffered from this skewness. A formal evaluation may require novel statistical methods that can represent and quantify the skewness of a multivariate distribution appropriately. Our demonstration suggests that, despite the seminal work by Gerlach, it is still an open question whether the distribution of personality should be characterized as categorical, dimensional, or their intermediate.

## Data Availability Statement

The R script used for the simulation is available at: https://github.com/kkatahira/personality_skewness.

## Author Contributions

KK, YK, YY, and SS designed the research. KK conducted simulations and analyzed the data. KK and SS drafted the manuscript. YK and YY provided critical revisions.

### Conflict of Interest

The authors declare that the research was conducted in the absence of any commercial or financial relationships that could be construed as a potential conflict of interest.
