# Supplementary material for: Commentary: A robust data-driven approach identifies four personality types across four large data sets
Source: Front Big Data. 2020 Feb 25;3:8. doi: 10.3389/fdata.2020.00008 (PMC7931870; doi:10.3389/fdata.2020.00008)
Supplement: Supplementary file 1 [file Data_Sheet_1.pdf]

Supplementary Text 1: Procedures of analysis in Gerlach et al. (2018)

Here, we briefly summarize the analysis procedure adopted by Gerlach et al. (2018). First, from responses to the questionnaires, five latent dimensions which correspond to the five main domains of the Big Five model are extracted by exploratory factor analysis (Cattell, 2012), assuming the orthogonality of the factors. The five-factor scores for each respondent are then estimated. By these scores, each respondent is localized in a five-dimensional (D) space of personality.

Next, to identify clusters in the distribution of factor scores, Gaussian mixture models (GMM) are fitted to the estimated five-factor scores. GMM represents a given distribution by a weighted average of a finite number of Gaussian (normal) distributions. Each Gaussian component is a candidate of a single cluster. The number of Gaussian components is determined from data using the Bayesian information criterion (BIC), a common measure for statistical model selection. A smaller BIC indicates a more probable model.

The Gaussian components are then verified as to whether they are truly meaningful clusters. To achieve this, the probability densities at the centre of each component in a 5D space are estimated using the kernel density estimation. The estimates are compared with 'a null model' that corresponds to the density function averaged over estimated densities from 1,000 randomized (shuffled) data sets, which destroys the relationship between the five factors. The clusters whose centres have a significantly higher density ($p < 0.01$, and the density ratio, called the 'enrichment' is more than 1.25) compared with the null model are deemed as meaningful clusters.

Supplementary Text 2: Simulation procedure

By using the synthesized data set, we illustrate how Gerlach et al.'s analysis works under two scenarios (**Figure 1** of the main text). Here, we consider 2D space, rather than 5D space as in Gerlach et al., for illustrative purposes. For the case with cluster structures, we generated samples from GMM with three components, whose means were (dimension 1, dimension 2) = (0, 2), (-1.5, -0.5), and (1.5, -0.5) and all the variance–covariance matrices were diagonal matrices whose diagonal components were 0.5. The mixing proportions were 0.4, 0.3, and 0.3, respectively. The resulting samples were standardized such that each variable has zero mean and unit variance.

For the case with a skewed distribution, we first generated 100,000 samples from two independent skew-normal distributions with parameters (Azzalini, 2005): $\xi = -1, \omega = 1, \alpha = 4$ (for the first variable) / -4 (for the second variable; **Supplementary Figure 1A**). These parameter sets result in distributions with skewness of 0.796 and -0.796. Thereafter, the samples were rotated by 45° (**Supplementary Figure 1B**). This rotation is crucial for observing spurious clusters that are deemed meaningful, because the test of meaningful clusters is performed by evaluating the dependence

between multiple factors, which are compared to the product of the marginal distribution (assuming independence). If all the factors are mutually independent, no meaningful cluster is observed irrespective of the shape of the marginals. By construction, the generated data have no (multiple) cluster structure.

We fitted GMMs to the generated samples (**Figures 1B, D** of the main text). For the case with a cluster structure, we found that the model with three Gaussian components is appropriate (it gave the minimum BIC). For the case with a skewed distribution, we found that the model with seven Gaussian components is appropriate.

Next, we examined whether each Gaussian component obtained by GMM is a meaningful cluster. We constructed a null model by re-assigning the factors across samples (**Supplementary Figure 1C**; here, only the case with a skewed distribution is shown). We note that the density surface of the null model has a shape different from that of the original data (**Supplementary Figure 1B**). This is because the skewness of the synthesized data was embedded in the joint distribution of two variables by rotation: the shuffling procedure destroyed this dependence. As a result, there were regions where the density of the original data was significantly higher than the null model, which gave higher values of enrichment and smaller *p*-values (**Supplementary Figure 1E**). In this case, the centre of the three components fell into these regions. These three components are identified as ‘meaningful clusters.’

Supplementary Text 3: Evaluation of the skewness of factor scores for Jonson-300 dataset.

To examine if the factor scores analyzed by Gerlach et al. (2018) are skewed, we evaluated the skewness of each factor for the Johnson-300 dataset (Johnson, 2014). Specifically, we analyzed the factor scores provided by Gerlach et al. (https://github.com/amarallab/personality-types/tree/master/data_filter; the code for generating this data is also available at the same repository). **Supplementary Figure 2** presents the density plot for each factor. We computed the skewness of the distributions of each factor. A negative (/positive) skewness indicates that the distribution is left (/right)-skewed. We also evaluated the 95% confidence intervals (CIs) of skewness, based on 1000 bootstrapped samples. It is noteworthy that none of the CIs contained zero (symmetric distribution), indicating that the marginal distributions of all five factors are significantly skewed.

Figures

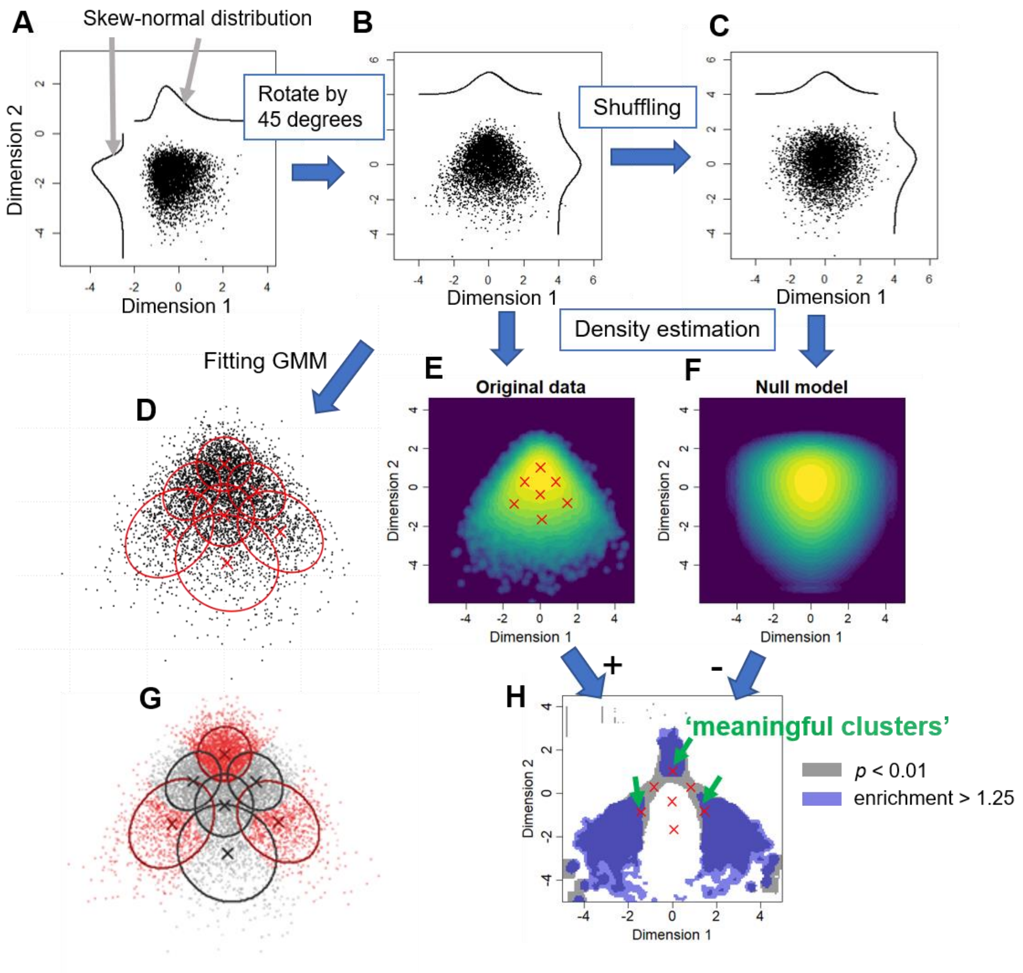

Supplementary Figure 1 | How a spurious 'meaningful cluster' emerges from a skewed distribution. **(A, B)** Synthesized data. The values of two variables are drawn from skew-normal distributions **(A)** and then rotated by 45° **(B)**. **(C)** The random null model is obtained by shuffling the values of each dimension (destroying the relationship between dimensions). **(D)** Gaussian mixture models. Red crosses indicate the centre (mean) of the Gaussian components. Red ellipses indicate the one standard deviation contours of the Gaussian components. **(E)** Log probability density of the original (synthesized) data obtained using kernel density estimation. **(F)** Log probability density of a random null model. **(G)** The samples classified into three meaningful clusters (red ellipses) are colored as red while other samples are colored as grey. **(H)** Evaluation of clusters. The Gaussian components whose means fall into grey-shaded regions ($p < 0.05$) and blue-shaded regions (enrichment > 1.25) are deemed 'meaningful clusters.' R-code to reproduce this plot can be found at https://github.com/kkatahira/personality_skewness.

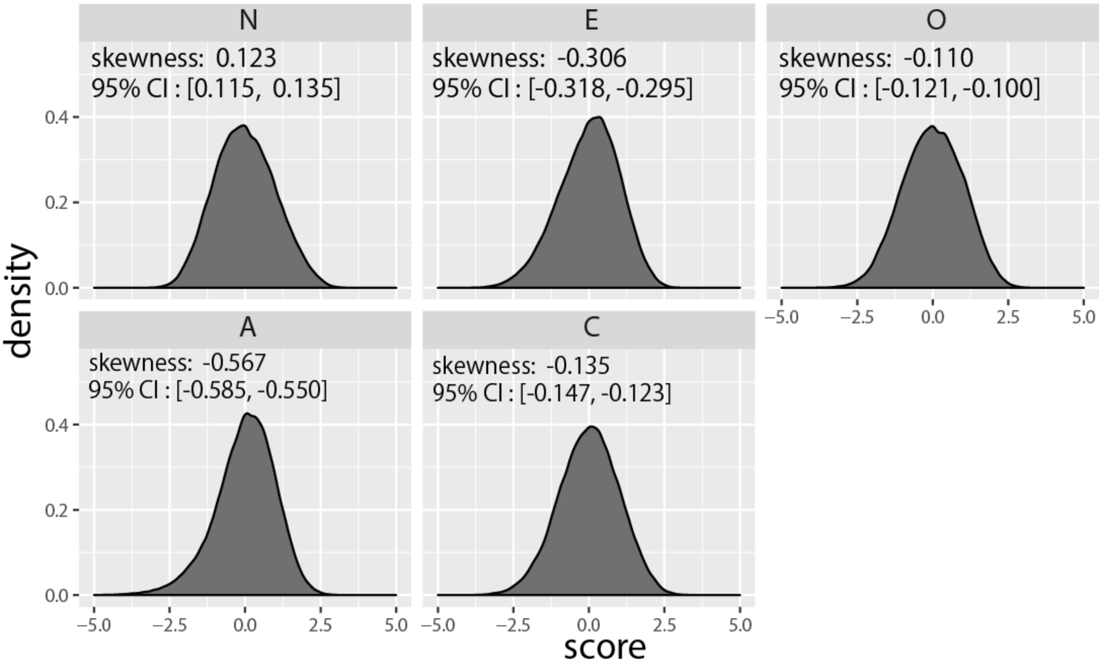

Supplementary Figure 2 | Skewness of the factor scores obtained from IPIP-300 data (from Gerlach et al., 2018). Density plots represent the marginal distribution of factor scores (N: neuroticism, E: extraversion, O: openness, A: agreeableness, and C: conscientiousness). The skewness of the distributions and their 95% confidence intervals (CIs) obtained via the bootstrap method are shown for each factor.

References

Azzalini, A. (2005). The skew-normal distribution and related multivariate families. *Scand. J. Stat.* 32, 159–188.

Cattell, R. (2012). *The scientific use of factor analysis in behavioral and life sciences*. Springer Science & Business Media.

Gerlach, M., Farb, B., Revelle, W., and Nunes Amaral, L. A. (2018). A robust data-driven approach identifies four personality types across four large data sets. *Nat. Hum. Behav.* 2, 735–742. doi:10.1038/s41562-018-0419-z.

Johnson, J. A. (2014). Measuring thirty facets of the Five Factor Model with a 120-item public domain inventory: Development of the IPIP-NEO-120. *J. Res. Pers.* 51, 78–89. doi:10.1016/j.jrp.2014.05.003.
